# Reducing Video Verification Burden: Machine Learning Classification of Head Acceleration Events in Youth Football

**DOI:** 10.21203/rs.3.rs-9368934/v1

**Published:** 2026-05-20

**Authors:** Giovanny A. Romero A., Josh Cherian, N. Stewart Pritchard, Joel D. Stitzel, Lyndia C. Wu, Ryan S. McGinnis, Jillian E. Urban

**Affiliations:** Wake Forest University School of Medicine; Wake Forest University School of Medicine; Wake Forest University School of Medicine; Wake Forest University School of Medicine; University of British Columbia; Wake Forest University School of Medicine; Wake Forest University School of Medicine

**Keywords:** Head acceleration events, youth American Tackle football, machine learning classification, Video-based ground truth labeling

## Abstract

This study aimed to develop and evaluate a machine learning pipeline to classify head acceleration events (HAEs) in youth American football and reduce the manual burden of video verification. An eXtreme Gradient Boosting (XGBoost) classifier was trained on three seasons of instrumented-mouthguard data from three organizations, including athletes aged 11–14 years, using a comprehensive set of kinematics-derived features. Model performance was evaluated under a highly imbalanced outcome distribution using precision, recall, and F1 score, and confirmed via permutation testing to ensure results exceeded chance performance. The proposed feature set and model achieved comparable or superior performance relative to previously published support vector machine approaches, while operating under more heterogeneous, real-world field conditions. To quantify operational burden, we compared the manual effort required to review all sensor-recorded events versus only those flagged as potential HAEs across resultant linear acceleration review thresholds. Total video review time was reduced from approximately 90 hours (all events reviewed) to under 8 hours (classifier-flagged events only), a 91% reduction in workload, while maintaining a misclassification rate of 2.5–3% of all events. When applied in conjunction to the model, thresholds in the 10–15 g range cut review time by more than half relative to reviewing all events and retained a low error in the model (4%). These findings show that an appropriately tuned classification and review-threshold pipeline can make large-scale video-verified monitoring of youth football HAEs substantially more feasible for research and surveillance applications.

## Introduction

Contact and collision sports inherently involve exposure of athletes to frequent concussive and non-concussive head impacts.^1–4^ In the U.S, contact sports account for approximately 45% of emergency department visits for sports-related traumatic brain injuries (TBIs) and concussions in children ages 17 and under.^5^ American football has one of the highest rates of head injuries, with a concussion incidence rate of 35.3 per 100,000 athlete-exposures, as well as a substantial number of repetitive head impacts sustained during both practices and games.^6,7^ Exposure to these impacts, even in the absence of concussion, has been shown to negatively affect brain health and increase brain injury risk.^8–11^ Due to the high incidence of both concussive and non-concussive impacts, American football has become a focal point for research on head impact safety, injury prevention, and long-term cognitive health outcomes.^1,12–14^

To better understand the risks associated with head impacts, researchers have developed methods to quantify exposure to head acceleration events (HAEs), providing an approximate measure of the frequency, magnitude, and distribution of impacts experienced by athletes during sport participation.^1,14–17^ Wearable sensors, such as instrumented mouthguards and helmet systems, enable real-time HAE capture in sports such American football.^1,12,18^ These sensors provide valuable insights into injury mechanisms and inform head injury reduction strategies.^19–21^

Wearable sensors used in instrumented mouthguards or mouthpieces are often configured to trigger when the accelerometer exceeds a prescribed threshold, capturing events associated with true HAEs (i.e., true positives) and incidental events (i.e., false positives), requiring verification to ensure accuracy.^22^ For example, in American football, players are not actively in play for the entire duration of the game, which can lead to the collection of false HAEs using instrumented mouthguards (i.e., chewing, decoupling).^23^ In a study from Marks *et al*.^12^ approximately 9% of the total 57,988 mouthpiece-triggered events, recorded using a 5 g linear acceleration trigger and a 50 ms recording window, were considered true HAEs after undergoing rigorous video verification.^12^ Research utilizing other wearable sensors, such as the Head Impact Telemetry (HIT) system, has implemented threshold-based filtering of linear acceleration to identify true impacts.^24,25^ Best practices outlined by the Consensus Head Acceleration Measurement Practices (CHAMP) suggest that video-verification of HAEs should be implemented to confirm sensor recorded events.^22^ Numerous studies have utilized this method to ensure data accuracy and reliability.^26^ However, this method can be time-consuming, especially when dealing with a large number of HAEs.

Several studies have implemented other methods to distinguish between true and false HAEs. Computational approaches, such as machine learning (ML), have been proposed as a parallel workflow with video verification to reduce the time burden of HAE classification.^23,27–29^ Wu *et al*.^23^ demonstrated approximately 91% recall using a Support Vector Machine (SVM) to identify true HAEs from instrumented mouthguards in football.^23^ Similarly, Goodin *et al*.^29^ reported ~ 94% recall with extreme gradient boosting (XGBoost) for Australian football HAEs.^29^ These studies highlight the potential of ML algorithms to automate HAE classification and reduce the need for labor intensive video verification while maintaining accuracy in HAEs classification. However, these studies are limited by the relatively small datasets used for model development. For instance, Wu *et al*.^23^ used instrumented mouthguards on 7 collegiate and 3 youth football players across 38 player-sessions (15 games, 23 practices). Similarly, Goodin *et al*.^29^ relied on data from five players on one college team during one season.^29^ In both cases, the data used for model development and evaluation had similar representation of true and false HAEs, with Goodin *et al*. down sampling the data set to match classes, and neither approach reflects the substantial class imbalance typically observed in real-world data and that could impact model performance.^23,29^

Building on this prior work, this study aimed to develop a robust ML technique for classifying HAEs, using youth football as the primary source of training and validation data. A total of 92,832 potential impact events were collected across five teams and 84 player-seasons of male athletes (11–14 years old) over multiple seasons, encompassing thousands of athlete-exposures and extensive gameplay data (141 practices, 45 games).^1,12^ A comprehensive ML pipeline was developed, incorporating a robust biomechanical feature set and multiple classification algorithms to improve event detection accuracy. The proposed framework has the potential to reduce manual video verification by about 90%, turning 10 hours of video review into roughly 1 hour, while preserving classification performance for true HAEs.

## Methods

### Study Sample

Impacts were recorded across three seasons from five youth American football teams (2021, *n* = 2 teams; 2022, *n* = 1 team; 2023, *n* = 2 teams) across three organizations participating in a local youth football league operating under the guidelines of American Youth Football (AYF). All teams practiced and played games in the Piedmont Triad region of North Carolina, USA. Across all seasons, data were collected from 84 male athletes (11–14 years) who participated in a total of 141 practices and 45 games. This study followed the strengthening the reporting of observational studies in epidemiology (STROBE) guidelines for collecting and reporting data.^30^ The Wake Forest University School of Medicine Institutional Review Board (IRB) approved the study protocol. Written informed consent from parents/guardians and participant assent were obtained from all athletes. All methods were performed in accordance with the relevant guidelines and regulations. Mean practice time was 103.12 ± 24.44 minutes per session, and mean game time was 92.4 ± 19.83 minutes per session. Data were previously video verified using best practices defined by the CHAMP guidelines.^22^ Sensor motion data were time synchronized with video, and researchers systematically reviewed all mouthguard recordings in parallel with video, classifying each event as a true or false impact and documenting additional details for every verified impact.^1,12,22^ The number of verified true impacts per team varied from 220 to 5182, reflecting differences in team size and the number of athletes enrolled in the study, as well as season length and practice activities.^1^ Corresponding false impact detections ranged from 1,977 to 29,052 per team, leading to imbalance ratios between 4.99:1 and 16.66:1.Detailed information is presented in Table 1.

### Instrumentation

Athletes were equipped with a personalized mouthpiece containing an embedded triaxial accelerometer and gyroscope.^31^ This custom-designed mouthpiece was created for each athlete based on an oral optical scan (3shape, Copenhagen, Denmark). Data collected for the custom-designed mouthpiece were processed following the procedures outlined by Rich *et al*.^31^ Calibration of the data was performed, converting it into units of g (linear acceleration) and rad/s (rotational velocity). Table 2 summarizes the specifications for data collection across the different seasons. Across the multiple seasons, two different data-capture configurations were used. To ensure consistency and enable direct comparison of impacts, a standard configuration was defined. In this configuration, data are resampled at 3,200 Hz, and each recorded event consists of 10 ms of data preceding the peak detection point (32 samples) and 40 ms of data following this point (128 samples), yielding a total of 160 samples centered on the maximum acceleration for every impact. However, for all seasons, the trigger threshold was set at 5 g on any single axis for a minimum duration of 3 ms.

### Data Classification

**Figure 1:**
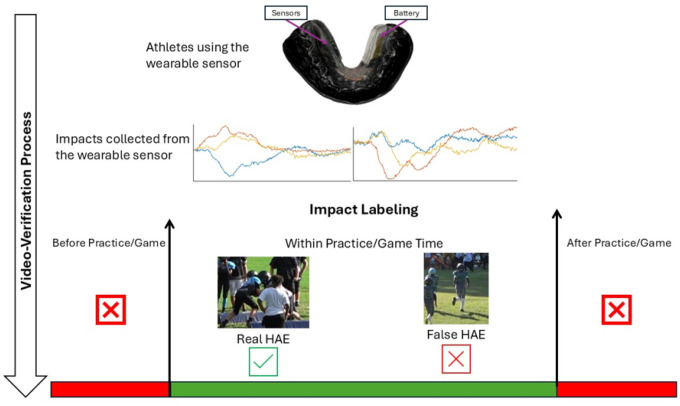
Event classification guideline

All data recordings used in this study were manually verified with video, following the best practices outlined by CHAMP.^22^ For each data collection session (i.e., game, practice), both the start and end times were recorded. Additionally, athletes’ attendance and mouthguard usage were documented for every session. Because each event includes an associated timestamp, any data recorded outside of session times were labeled as “not active”. Furthermore, if an athlete chose not to wear the mouthguard during a session, this was noted as “not worn.” Consequently, all recordings identified as either inactive or not worn were excluded from the final analysis. If the event was labeled as active and worn a further video verification was performed to validate if the event was true (i.e., associated with an HAE) or false; true events underwent detailed review to visualize and document the specific impact characteristics, enhancing labeling confidence. This event hierarchy can be observed in Fig. 1. This labeling resulted in a total of 93,353 HAEs, of which 81,647 (87%) were labeled as false HAEs and 11,706 (13%) as true HAEs.

### Features

Linear acceleration (in g) and rotational velocity (in rad/s) signals, including measurements from all three axes and their corresponding resultant vectors, were utilized for feature extraction. In total, 267 distinct features were computed from each HAE. Many of these features correspond to commonly reported head kinematic characteristics, such as linear and rotational accelerations, velocities, and related temporal descriptors, that are widely used to quantify and describe head motion in biomechanics research.^1,12^ Other features were selected based on time-domain, frequency-domain, time-frequency characteristics and biomechanical risk metrics. A comprehensive description of each feature is provided in the supplemental digital content (Table A1) to facilitate detailed replication and interpretation of the analysis.

#### Biomechanical Risk Metrics

Individual risk values of each event were calculated. Linear and rotational acceleration measures were used to compute the linear risk (LR) and rotational risk (RR), which represent probabilistic estimations of concussion likelihood derived from translational and angular kinematics, respectively.^32,33^ To capture the combined contribution of these mechanical components, the combined probability of concussion (CP) model was applied, integrating peak linear and rotational acceleration.^34^ The youth combined probability of concussion (CPyouth) metric, specifically calibrated for pediatric head impact biomechanics, was computed as an event-level risk value for each individual.^35^ Additionally, a rapid maximum brain strain estimate was calculated using a second-order system approach (DAMAGE), enabling efficient assessment of brain tissue deformation associated with head impacts.^36^ These risk metrics were incorporated as additional features to place impacts on a common, interpretable injury-severity scale and to connect the model’s kinematic inputs to established concussion risk functions and brain tissue response models.

#### Frequency-Domain Features

Amplitude spectral density (ASD) in decibels was computed for the resultant linear acceleration and angular velocity signals using a fast Fourier transform (FFT). The resultant signal was derived by combining the three axis components (x, y, and z) of each kinematic measure through a vector magnitude calculation. No additional filtering was applied prior to frequency-domain analysis. For each resultant signal, ASD values were sampled at regular frequency intervals (every 50 Hz) up to the Nyquist frequency of 1600 Hz, with interpolation applied where necessary to ensure consistent feature extraction across signals.

#### Time-Frequency Features

A continuous wavelet transform (CWT) based approach was used to generate a time-frequency representation of each resultant linear acceleration and rotational velocity signal. The frequency spectrum was divided into consecutive 200 Hz bands up to the Nyquist frequency, and within each band, the maximum amplitude of the wavelet coefficients was determined, capturing the most prominent transient events or peaks within each frequency range over the course of the signal.

#### Time-Domain Features

In the time domain, for each signal, peak-based features (maximum absolute value, peak-to-peak amplitude, and root mean square amplitude) summarize intensity, variability, and average energy over the impact interval. The frequency of sign changes is quantified via zero crossings and zero-crossing rate, and abruptness of change is captured by the maximum absolute first difference (jerk for acceleration and maximum rate of change for rotational velocity). The area under the curve is computed as a measure of the cumulative effect of the signal, sample entropy is used as an index of signal irregularity, and low, median, and high quantiles (5th, 50th, and 95th percentiles) describe the distribution during the impact. Temporal structure is further characterized by the time to the first peak, the average absolute slope, slopes in short windows around the main peak, and segment-specific areas under the curve obtained by dividing the time series into ten equal segments to indicate when during the trial most of the motion occurs. To capture more subtle waveform properties, the Higuchi fractal dimension is calculated for each original time series as a non-linear index of temporal complexity. Finally, the second derivative is used to identify inflection points, enabling detection of intervals where the signal is strong and exhibits pronounced changes in curvature.

### Model Training and Evaluation

Algorithms explored for modeling the relationship between signal features and HAEs include K-Dimensional (KD) tree, logistic regression, random forest, SVM, decision tree, K-Nearest Neighbors (KNN), binary Kernel classification, and eXtreme Gradient Boosting (XGBoost). A 5-fold stratified group cross validation was implemented to evaluate model performance, ensuring that each fold preserved the overall class distribution while keeping all observations from the same participant within either the training or test split. For each fold, data were split with shuffling; feature standardization was performed using statistics (mean and standard deviation) computed from the training data only, which were then applied to transform the corresponding test data; and models were trained with a class imbalance weight computed from the training labels. ML models were implemented and evaluated in Python 3.12.7 (Anaconda) using scikit-learn 1.5.1 and XGBoost 2.1.1.

Permutation testing was conducted on the best performing model to assess whether its performance exceeded what could be obtained by chance. For each permutation, the training labels were randomly shuffled while keeping the feature matrix, test labels, model hyperparameters, and class-imbalance weighting fixed; the model was retrained on the permuted training data, and performance metrics were recomputed on the held-out test set. Performance was evaluated using metrics such as precision, recall, F1-score, and area under the receiver operating characteristic curve (AUC).

In addition to permutation testing, we also implemented the model described in Wu *et al*. as a comparator.^23^ Here, we implemented the same features and modeling approach (SVM), but trained and evaluated the model within the modeling framework described above. This model serves as the primary comparison due to its direct relevance to American football's same sport environment, featuring comparable impact mechanics, equipment, and player dynamics, making it more applicable than a model from studies of other contact sports.

### Feature evaluation

The XGBoost model enables assessment of feature importance after training, allowing identification of the most influential predictors. Using these importance rankings, an additional analysis was conducted in which features were added sequentially in order of importance, beginning with the single most influential feature and then iteratively including the next most important features. For each subset of selected features, the data were split into 70% training and 30% testing using a stratified group split that preserved the class distribution while ensuring that all observations from a given participant were contained entirely in either the training or the test set, and model performance was recorded for each feature subset.

### Post-Hoc Analysis of High Magnitude Events

Given that high-magnitude events influence biomechanical risk metrics and may be closely associated with potential brain injury,^32,34,37^ a separate threshold-based analysis was conducted on the test dataset. This evaluated impacts exceeding specified resultant linear acceleration values, to determine how different cut-points affect both model performance and the volume of events requiring manual review (i.e., video verification). Impacts included at each threshold were defined as those with resultant linear acceleration greater than the specified threshold value. Thresholds were defined using percentiles of the resultant linear acceleration distribution, starting at the 5th percentile and increasing in 5-percentile increments up to the 95th percentile. This threshold analysis was performed for the top-performing and comparator model to identify an optimal acceleration cutoff that minimizes misclassification of true impact events (i.e., false negatives).

### Time-Savings Analysis

In addition to evaluating how high-magnitude events influence biomechanical risk metrics, a separate analysis examined their impact on the time required to review events that the model classified as true, assuming an average of 30 seconds of video review per impact. Review time was quantified at multiple high-magnitude thresholds to assess how different cut-points trade off reductions in manual verification time against the need to retain and inspect higher-magnitude events that may carry greater injury risk. To further characterize the operational burden of uncertainty, a symmetric window around the conventional classification threshold of 0.5 was applied to model-predicted probabilities, and review time was recalculated for HAEs falling within this low-confidence region.

## Results

### Model Selection and evaluation

Among the classification models evaluated, the XGBoost algorithm consistently demonstrated superior performance relative to the other ML models. XGBoost achieved the highest values for recall and precision, indicating more effective discrimination between true and false events. Additionally, XGBoost outperformed the other models across key metrics, including precision, F1-score, and AUC, reflecting its robustness in identifying true HAEs within the dataset. Table 3 provides a detailed comparison of each classifier’s performance across all evaluation metrics, underscoring the consistent advantages of XGBoost in this context. Permutation testing showed that model performance was statistically significantly better than random chance for recall, precision, F1 score, and AUC (p < 0.01). To provide a direct comparison with previously reported methods, the feature set described by Wu *et al*.^23^ was implemented and evaluated using a SVM classifier following the same algorithmic parameters (C and kernel type) reported in their study.^23^ In that work, the SVM achieved overall recall and precision values exceeding 0.88 on both cross-validation and an independent youth dataset, with corresponding overall F1 scores above 0.88; those results were obtained using 387 impacts in the cross-validation cohort and 32 impacts in the independent youth cohort.^23^

### Feature evaluation

Adding features in order of importance led to progressive improvements in model performance. Using only the single most important feature (Diffuse Axonal, Multi-Axis, General Evaluation (DAMAGE)), the model achieved modest discrimination (F1 0.25, recall 0.54, precision 0.16, AUC 0.66). As additional features were incorporated, first the time-frequency metric (Peak magnitude of CWT coefficients (0–200 Hz in 200 Hz bands) for resultant rotational velocity), then Higuchi fractal dimensions, average slopes, sample entropy measures, and area under the curve and spectral density features for linear and rotational kinematics, performance improved steadily, with recall increasing to 0.75, precision to 0.86, F1 to 0.80, and AUC to 0.93 when the top 20 features were included. The most important features identified in the model illustrate an approach to analyzing biomechanical signals related to HAEs. Table 4 summarizes the 20 most influential features and their corresponding performance metrics.

### Post-Hoc Analysis of High Magnitude Events

**Figure 2:**
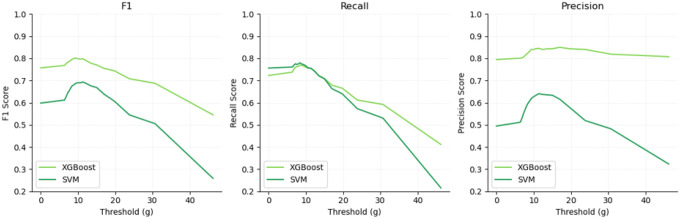
Performance comparison of XGBoost model and comparator model across different g thresholds for F1 score recall, and precision.

A separate threshold-based analysis was conducted on the test dataset, where impacts exceeding specified resultant linear acceleration thresholds were selected and classified using the trained models to evaluate how cut-points affect performance and video verification workload. Threshold values ranged from 0 g, which represents inclusion of all events, up to 46.23 g at the 95th percentile. Values at the 5th, 25th, 50th, 75th, and 90th percentiles were 6.38 g, 7.99 g, 10.57 g, 17.00 g, and 30.69 g, respectively. The corresponding percentile-specific g-thresholds are reported in Table A2. Across the high magnitude evaluation, recall decreased as the threshold increased, declining from 0.75–0.77 at the lowest thresholds to approximately 0.10 at the highest thresholds in the comparator model, and to approximately 0.61 in the XGBoost model. Precision showed the opposite pattern, improving from about 0.60–0.65 at low thresholds to 1.0 at the highest thresholds in the XGBoost model, but dropping to 0.2 with the comparator model. Consistent with these trends, F1 scores were highest at the lowest thresholds (0.75–0.77), decreased at mid-range thresholds, and approached zero at the highest thresholds in the XGBoost model, while exhibiting a steadier decline across thresholds in the comparator model. Performance across the different thresholds is illustrated in Fig. 2.

### Time- Savings Analysis

**Figure 3:**
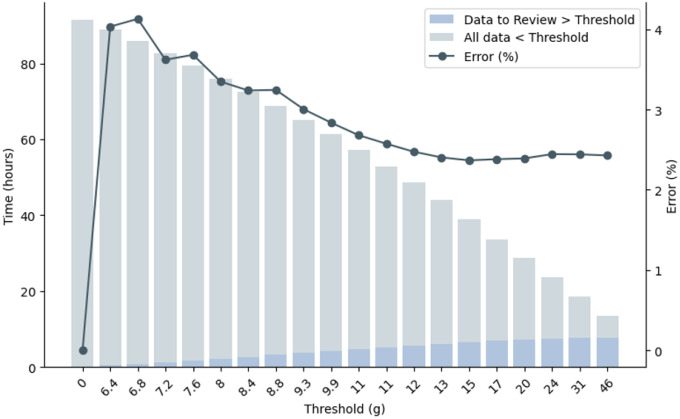
Time in hours required to review XGBoost model–predicted events (true positives + false positives) for impacts below threshold (blue) and all impacts exceeding threshold (gray); dotted line indicates misclassification error (false negatives as % of all impacts) at each threshold.

Continuing with the threshold framework, the analysis establishes that events exceeding specified acceleration thresholds should undergo manual video verification and be excluded from automated model assessment, while lower-magnitude events should be classified by XGBoost. At low thresholds (< 11 g), few model-flagged events require review (low volume), but total review time remains high because most impacts exceed these thresholds and require manual evaluation. As the threshold increases, review time for model-flagged events below the threshold rises, but total verification workload decreases substantially since fewer high-magnitude impacts require manual review. The proportion of false negatives among model-flagged events decreases with increasing threshold, indicating that fewer true high-magnitude events are missed when review is prioritized for only the highest-magnitude impacts. Figure 3 illustrates these patterns by showing, across thresholds, the total hours required to review model-flagged events below and all impacts above each threshold, along with the corresponding misclassification error (false negatives as a percentage of all verified impacts).

**Figure 4:**
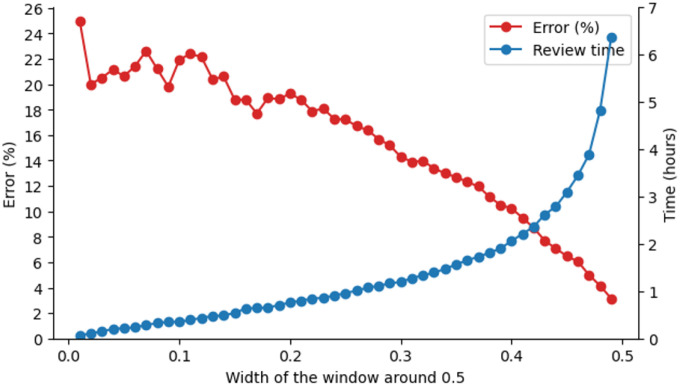
Error and review time as a function of the low-confidence window around the classification threshold. Red line shows the misclassification error within the low-confidence region (false positives/ HAEs within the window). Blue line shows the total review time required to manually verify HAEs within this window (true positive plus false positives).

A symmetric window was defined around the conventional 0.5 decision threshold used to convert predicted probabilities into binary classifications. Within this low-confidence window, there is a clear trade-off between review time and classification error. As the width of the low-confidence window increased from 0 to 0.5, review time increased steeply from less than 1 hour to more than 6 hours, while the error rate for HAEs within this window (false positives divided by the number of HAEs within the window) declined from about 25% to below 5%. At intermediate window widths (e.g., 0.3–0.4), review time remained around approximately 3–4 hours, with error reduced to 10–15%, indicating that relatively narrow low-confidence regions can capture most misclassified HAEs while limiting additional review workload. Detailed results are presented in Fig. 4.

## Discussion

The findings of this study demonstrate the potential of ML approaches to enhance the detection and classification of HAEs in youth football. By analyzing a large dataset of impacts recorded by mouthguard wearable sensors and developing a novel feature-based classification pipeline, this study provides evidence that automated methods can substantially improve the efficiency and consistency of data verification processes. These results highlight the feasibility of using computational models to substantially reduce the manual review burden for HAEs, thereby streamlining the classification workflow while maintaining accurate identification of impacts that warrant closer inspection.

During permutation evaluation, the low p-values observed for precision, accuracy, sensitivity, balanced accuracy, F1 score, and AUC (< 0.01) indicate that the model’s predictive performance is statistically significantly better than random chance, confirming that it captures meaningful patterns in the data and supporting its validity. Coupled with the mean metrics observed during cross-validation, precision 0.73 ± 0.03, AUC 0.90 ± 0.02, and recall 0.65 ± 0.07, suggest that the model has reasonably good capacity to distinguish true impact events, with evidence of useful discrimination between classes. Although recall is somewhat lower, it still reflects reasonable detection of true positives given class imbalance challenges, and the F1 score of 0.69 ± 0.05 demonstrates a solid trade-off between precision and recall. These combined results affirm the model’s efficacy in detecting true impact events, highlight its robustness and stability, and suggest it is unlikely to overfit the training data. This strengthens confidence in the model’s application to real-world scenarios, where balancing false positives and false negatives is vital for practical utility, particularly in reducing the burden of manual review for biomechanical event prediction.^38,39^

The most important features identified in the proposed model help clarify why its performance diverges from previously published pipelines. While prior work has highlighted the value of frequency-domain and wavelet-based features alongside time-domain peak kinematics and summary variables,^23,29^ the present feature set extends this by emphasizing frequency-resolved rotational dynamics, nonlinear signal complexity, and injury-oriented biomechanical metrics that more directly capture the irregular, transient nature of real-world head impacts.^32,34,40^ For example, continuous wavelet–based features of resultant rotational velocity across 0–400 Hz target frequency content shown to be relevant to head impact response, whereas nonlinear descriptors such as Higuchi fractal dimension and sample entropy characterize signal complexity beyond what can be represented by peak magnitudes or simple integrals alone.^40,41^ In contrast, previous feature sets rely more on conventional linear kinematic summaries, such as peak linear acceleration, peak rotational velocity, and simple time-domain averages or integrals of these signals, which may fail to capture subtle differences between true HAEs and non-impact artifacts in on-field data.^23,29^ By refining the feature space toward variables that integrate temporal, frequency, nonlinear, and brain-strain–oriented information, the current model appears to capture aspects of impact mechanics that were either under-represented or not systematically leveraged in previous implementations. This is aligned with prior reviews that suggest that systematically exploring a broad range of kinematic and biomechanical signal features can help identify derived variables with improved discriminatory ability, and may inform future, more domain targeted feature engineering efforts.^42^

This study highlights important limitations of existing HAE classification pipelines when deployed under realistic, large-scale field conditions, and demonstrates the value of targeted feature selection under severe class imbalance. Wu et al.^23^ originally reported strong performance using 81 sensor-derived features and an SVM for HAE detection in a collegiate sample (n = 387 impacts, plus an independent youth set of n = 32), following infrared proximity classification to select high-proximity events, establishing that approach as a reference for subsequent work. In the present study, the Wu feature set was reimplemented within our pipeline and evaluated using an SVM configured to match their reported parameters, but only on high-confidence verified impacts and non-impacts (resulting in a more balanced subset), and performance was lower than originally reported. This discrepancy may reflect differences in sample composition and size, sensor hardware and mounting, dataset filtering (high-confidence labels only), the absence of their infrared pre-classification step, and the evaluation of different classification models (XGBoost rather than SVM), all of which could reasonably influence generalizability and observed performance.^23^ In contrast, the current model was trained on three seasons of data from three organizations involving athletes aged 11–14 years and operated under a substantially higher class imbalance ratio (6.93:1), a setting in which many machine-learning algorithms typically struggle to maintain balanced precision and recall.^43^ Even in this more challenging context, the model achieved comparable or superior precision, recall, and F1 scores using 20 features that were selected empirically for their discriminative performance, rather than hand-designed a priori. These findings, together with the reevaluation of the Wu feature set, suggest that model architectures and feature sets developed under more controlled or homogeneous conditions may not translate directly to large, heterogeneous youth sport cohorts, underscoring the importance of rigorous external validation under realistic operating conditions before adopting HAE classification algorithms for widespread surveillance or risk monitoring.

One of the primary objectives in developing the model was to differentiate between true and false events, thereby reducing the volume of data requiring manual video verification while still ensuring that high-magnitude impacts receive appropriate scrutiny. High-magnitude events cannot be disregarded because they strongly influence biomechanical injury risk metrics and cumulative exposure;^32,34,37^ misclassification of these impacts could substantially distort exposure and risk estimates over a season, highlighting the need for accurate identification and review of such critical events. To address this, in the proposed workflow, all events exceeding a specified resultant linear acceleration threshold (e.g., 10–15 g) are automatically routed for manual video verification and are not classified by the model, whereas events below this threshold are first classified by the XGBoost model and only model-flagged positives (putative HAEs) undergo review. This design explicitly acknowledges that model performance declines at higher magnitudes and avoids relying on automated classification for the impacts that contribute most heavily to risk metrics. This review-time analysis demonstrated that using the proposed pipeline reduced the burden of manual verification across all magnitude thresholds compared with reviewing every recorded impact; for example, when all events were considered, total review time decreased from nearly 90 hours to under 8 hours when only model-flagged events (true positives plus false positives) as potential HAEs were reviewed. As the g threshold increased, review time for model-flagged events declined steeply, whereas the time required to review all events also decreased but remained substantially higher. In practical terms, very low review thresholds (< 6–7 g) offer little benefit because nearly all impacts still require review despite only modest misclassification error. In contrast, review thresholds in the 10–15 g range cut review time by more than half while keeping the proportion of missed impacts low and relatively stable at approximately 2.5–3% of all impacts. The model itself can also be tuned to prioritize sensitivity or precision differently for various applications, for instance, contexts with lower tolerance for missed HAEs may favor lower thresholds (e.g., 10–12 g) or sensitivity-optimized model parameters, whereas resource-constrained settings may adopt higher thresholds (e.g., 15 g) or precision-optimized configurations, accepting a modest increase in undetected impacts in exchange for a further reduction in manual review burden and enabling real-time monitoring of high-magnitude events that could be associated with elevated biomechanical risk.^26,44^

The classification threshold analysis demonstrates a clear operational trade-off between review time and classification error within the low-confidence region around the 0.5 decision threshold. As the symmetric window around 0.5 widens, the time required to review events flagged by the model increases from near zero to more than 6 hours, reflecting the growing number of HAEs routed for manual inspection. At the same time, the error rate within this region decreases from roughly 25% to below 5%, indicating that broader low-confidence windows capture a larger proportion of misclassified events but at a substantial cost in reviewer workload. This pattern parallels threshold-selection trade-offs described in other machine learning applications, where improving operational efficiency by limiting review volume necessarily comes at the expense of higher error, and conversely, aggressive uncertainty handling reduces error but demands greater human effort.^45,46^ In the context of HAEs, these results suggest that the width of the low-confidence window should be chosen in accordance with clinical and surveillance priorities: narrower windows may be appropriate when minimizing review time is dominant, whereas wider windows are preferable when reducing classification error within the uncertain region is more important, even if this requires considerably greater review time.^44,47^

The current model faces several limitations that provide avenues for future improvement. First, the confidence of video-based event classifications serving as ground truth warrants discussion. These represent human judgments that, while thorough, are inherently subject to inter-rater variability and may not constitute perfectly reliable gold-standard labels. Variability in data recording conditions, such as sensor placement, environmental noise, or device calibration, may also influence feature calculation consistency and reliability.^23,29^ These variations can affect the model’s performance when applied across different settings or cohorts. Future work should investigate standardization protocols and feature extraction pipelines to mitigate these influences. Expanding model validation to diverse environments and populations will be crucial to ensure generalizability. Finally, ongoing enhancements targeting improving F1 score, recall, and precision will help better capture true events while limiting false events in athlete safety monitoring application.

This study presents an application of ML to classify American football-related impact events collected using instrumented mouthguards in a youth sports environment. This approach addresses an important need to improve impact detection accuracy while reducing reliance on time-consuming video verification in general sports settings. The results demonstrate that the model achieves strong predictive accuracy, recall, precision, and F1 performance, underscoring its potential to reduce manual review burdens while maintaining reliable identification of true impacts in an imbalanced setting. The study highlights the challenges of balancing recall and precision in such datasets and validates the model’s performance through permutation testing to confirm that the observed metrics are unlikely to arise by chance. In practical terms, the proposed review-threshold strategy reduces estimated manual video review time by more than half at moderate g-thresholds (approximately 10–15 g), while keeping the misclassification rate low and stable at roughly 2.5–3% of impacts, translating to several hours of reviewer time saved per season for each team at typical exposure volumes. By quantifying how misclassification affects summed biomechanical risk metrics and showing that most high-risk exposure is retained even when low-probability events are not reviewed, this work offers a concrete, pipeline framework for prioritizing which events require manual labeling review, thereby making athlete monitoring more feasible for research teams handling thousands of recorded impacts.

## Supplementary Files

This is a list of supplementary files associated with this preprint. Click to download.

- SupplementalDigitalContent.docx

## Figures and Tables

**Table 1:** Summary Data Distribution. Practice and game time reported in minutes.

Team	Athletes	Practices	Games	Practice Time per session (minimum, mean ± SD)	Game Time per session (minimum, mean ± SD)	True Impacts	False Impacts	Imbalance Ratio
2021 13U	17	25	9	103, 123.16 ± 13.50	54, 90.89 ± 19.46	721	4393	6.09
2021 12U	18	30	10	35, 95.07 ± 27.85	77, 91.90 ± 12.22	4390	29052	6.62
2022 13U	14	28	10	39, 83.18 ± 32.67	81, 99.80 ± 26.54	5182	25874	4.99
2023 13U	21	25	8	24, 100.14 ± 25.42	57, 86.62 ± 19.88	220	1977	8.99
2023 12U	14	33	8	56, 114.03 ± 22.75	60, 92.50 ± 17.06	1190	19830	16.66

**Table 2:** Data capture configuration

Conditions	2021–2022	2023	Standard
Sample frequency	4681 Hz	6400 Hz	3200 Hz
Samples before max point	70 samples (15ms)	96 samples (15 ms)	10 ms (32 samples)
Samples after max point	230 samples (49ms)	288 samples (45 ms)	40 ms (128 samples)
Total samples	300 samples	385 samples	160 samples

**Table 3:** Models Cross-Validation and Permutation Performance

Cross-Validation				
Model	F1 Mean ± SD	Recall Mean ± SD	Precision Mean ± SD	AUC Mean ± SD
KD Tree	0.654 ± 0.004	0.571 ± 0.004	0.765 ± 0.008	0.860 ± 0.003
Logistic Regression	0.497 ± 0.005	0.755 ± 0.009	0.371 ± 0.004	0.856 ± 0.005
Random Forest	0.630 ± 0.007	0.512 ± 0.006	0.821 ± 0.009	0.899 ± 0.006
SVM	0.657 ± 0.008	0.760 ± 0.006	0.578 ± 0.009	0.895 ± 0.004
Decision Tree	0.587 ± 0.006	0.636 ± 0.008	0.545 ± 0.008	0.749 ± 0.007
KNN	0.654 ± 0.004	0.571 ± 0.004	0.765 ± 0.008	0.860 ± 0.003
Binary Kernel Classification	0.657 ± 0.008	0.760 ± 0.006	0.578 ± 0.009	0.895 ± 0.004
**XGBoost**	**0.765 ± 0.005**	**0.713 ± 0.006**	**0.825 ± 0.005**	**0.931 ± 0.004**
SVM (WU)	0.568 ± 0.067	0.568 ± 0.067	0.478 ± 0.086	0.859 ± 0.0302
Permutation				
Model	F1 Mean ± SD (p value)	Recall Mean ± SD (p value)	Precision Mean ± SD (p value)	AUC Mean ± SD (p value)
**XGBoost**	0.01 ± 0.003 (< 0.01)	0.01 ± 0.002 (< 0.01)	0.12 ± 0.025 (< 0.01)	0.51 ± 0.014 (< 0.01)

**Table 4:** Feature evaluation by importance on the model

Feature Group	Feature	F1	Recall	Precision	AUC
Injury Risk	Diffuse Axonal, Multi-Axis, General Evaluation (DAMAGE)	0.25	0.54	0.16	0.66
Time–frequency	Peak magnitude of CWT coefficients (0–200 Hz in 200 Hz bands) for resultant rotational velocity	0.38	0.71	0.26	0.82
Nonlinear (time)	Higuchi fractal dimension for linear acceleration in the z axis	0.44	0.63	0.34	0.84
Time-domain	Average slope for linear acceleration in the y axis	0.68	0.70	0.67	0.90
Time-domain	Area under the curve sample 1 to 16 for linear acceleration in the x axis	0.69	0.73	0.66	0.92
Nonlinear (time)	Sample entropy for rotational velocity in the x axis	0.71	0.72	0.70	0.91
Time-domain	Average slope for linear acceleration in the x axis	0.71	0.71	0.71	0.92
Time-domain	Area under the curve sample 33 to 48 for resultant linear acceleration	0.71	0.71	0.71	0.92
Time-domain	Area under the curve sample 49 to 64 for resultant linear acceleration	0.75	0.72	0.78	0.93
Nonlinear (time)	Higuchi fractal dimension for linear acceleration in the y axis	0.77	0.73	0.80	0.93
Time–frequency	Peak magnitude of CWT coefficients (200–400 Hz in 200 Hz bands) for resultant rotational velocity	0.78	0.74	0.83	0.93
Frequency-domain	Amplitude spectral density values 50–100 Hz for resultant linear acceleration	0.79	0.74	0.84	0.93
Time-domain	Area under the curve sample 1 to 16 for linear acceleration in the y axis	0.78	0.72	0.84	0.93
Frequency-domain	Amplitude spectral density values 100–150 Hz for resultant linear acceleration	0.79	0.74	0.85	0.93
Nonlinear (time)	Higuchi fractal dimension for rotational velocity in the y axis	0.80	0.74	0.86	0.93
Time-domain	Root mean square amplitude linear acceleration in the y axis	0.79	0.74	0.86	0.93
Frequency-domain	Amplitude spectral density values 1200–1250 Hz for resultant linear acceleration	0.80	0.75	0.85	0.93
Time-domain	5 percentile value for resultant linear acceleration	0.79	0.74	0.86	0.93
Frequency-domain	Amplitude spectral density values 850–900 Hz for resultant linear acceleration	0.80	0.74	0.86	0.93
Frequency-domain	Amplitude spectral density values 200–250 Hz for resultant linear acceleration	0.80	0.75	0.86	0.93

## Data Availability

The data supporting the findings of this study will be made available through the Federal Interagency Traumatic Brain Injury Research (FITBIR) informatics system (https://fitbir.nih.gov/).
